# *Alu* Mobile Elements: From Junk DNA to Genomic Gems

**DOI:** 10.6064/2012/545328

**Published:** 2012-12-16

**Authors:** Sami Dridi

**Affiliations:** Nutrition Research Institute, The University of North Carolina at Chapel Hill, 500 Laureate Way, Kannapolis, NC 28081, USA

## Abstract

*Alus*, the short interspersed repeated sequences (SINEs), are retrotransposons that litter the human genomes and have long been considered junk DNA. However, recent findings that these mobile elements are transcribed, both as distinct RNA polymerase III transcripts and as a part of RNA polymerase II transcripts, suggest biological functions and refute the notion that *Alus* are biologically unimportant. Indeed, *Alu* RNAs have been shown to control mRNA processing at several levels, to have complex regulatory functions such as transcriptional repression and modulating alternative splicing and to cause a host of human genetic diseases. *Alu* RNAs embedded in Pol II transcripts can promote evolution and proteome diversity, which further indicates that these mobile retroelements are in fact genomic gems rather than genomic junks.

## 1. Introduction

*Alu* repeat elements are the most abundant interspersed repeats in the human genome. They are a family of short interspersed nuclear elements (SINEs) that use the reverse transcriptase and nuclease encoded by long interspersed nuclear elements (LINEs) to integrate into the host genome [1–3] and are found in the human genome in a number of ~1.100,000 copies, covering ~10% of its total length [4]. Functioning as transacting regulators of gene expression, pol III transcribed *Alu* and B1/2 (*Alu*-like elements in mouse) RNAs can interact with pol II and repress mRNA transcription [5–7]. Inverted *Alu* repeats are target for A-to-I editing by adenosine deaminases (ADARs) and can cause alternative splicing and drive proteome diversity [8]. Beside its role in human genomic evolution and diversity, *Alu* insertions and *Alu*-mediated unequal recombination contribute to a significant proportion of human genetic diseases [9]. *Alu* RNAs can also induce age-related macular degeneration following direct cytotoxicity to retinal pigment epithelium (RPE) cells [10].

In this brief paper, the author will describe the structure of human (*Alu*) and murine (B1, B2, ID, and B4) retroelements, a broad overview of the contribution of *Alu* retrotransposition to human diseases, and finally describe in depth a novel role of double-stranded *Alu* RNAs affecting the progression of age-related macular degeneration (AMD) and *Alu* editing by ADARs. 

## 2. Structure of **Alu ** and Murine Mobile Elements

**Figure 1:**
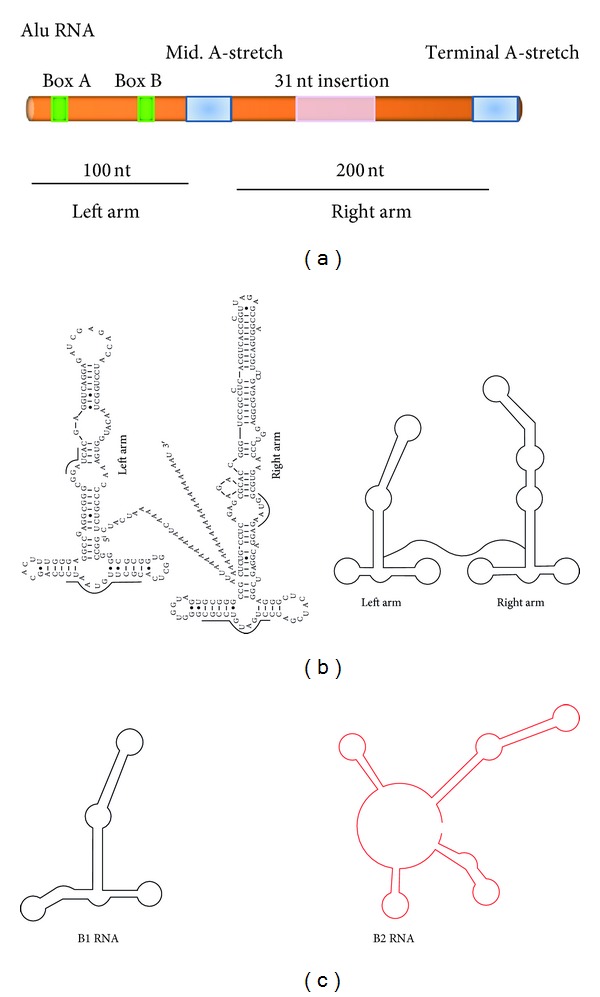
Architecture of *Alu*, B1, and B2 repeat elements. (a) *Alu* elements are about 300 nucleotides long composed of two arms joined by a mid. A-stretch and terminated by a poly (A) stretch. They contain two boxes (A and B) of the RNA polymerase III internal promoter. (b) *Alu* RNA secondary structure (adapted from [23]) and (c) B1 and B2 RNA secondary structure (adapted from [24]).

*Alu* typical sequences are ~300 nucleotides long and are classified into subfamilies according to their relative ages (for review see [11]). They have a dimeric structure and are composed of two similar but distinct monomers: left and right arms of 100 and 200 nucleotides long, respectively, held together by an A-rich linker and terminated by a short poly(A) tail (Figure 1(a)). Each of the *Alu* subunits originated from 5′ and 3′ terminal segments of 7 SL RNA [12–14]. *Alu* sequences contain internal Pol III promoter elements (Box A and Box B) and they are CG and CpG rich [11]. *Alu* subunits fold independently and conserve secondary structure motifs of their progenitor 7 SL RNA (Figure 1(b)). They were initially considered as selfish entities propagating in the host genome as “junk DNA” [15]. Now, it becomes more and more evident that the evolution of *Alu* subfamilies interacts in a complex way with other aspects of the whole genomic dynamics. *Alu* elements are specific to primates [11] and only one type of SINE in the human genome. The mouse genome contains four distinct SINE families: B1, B2, ID, and B4. B1 and B2 elements occupy approximately 5% of the mouse genome with about 550,000 and 350,000 copies, respectively [16]. Similar to *Alu*, B1 SINEs are also thought to be derived from 7SL RNA and transcribed by pol III into ~135 nucleotide B1 RNA, which approximates the left arm of *Alu* [17] (Figure 1(c)). B1 SINEs are monomers with an internal 29 nucleotide duplication [18]. Like the B1 element, B2 is transcribed by the polymerase III promoter sequence. Unlike B1, it shares significant homology at the 5′-end with tRNA, and they are believed to be derived from tRNA [19] and encode the ~200 nucleotide B2 RNA (Figure 1(c)) [20]. ID repeat elements are believed to be derived from a neuronally expressed BC1 gene, and they are 69 nucleotides long and are small in number about 42,200 copies; however, they have major presence in the rat genome [21]. The B4 repeat element appears to be a result of fusion of ID element at 5′-end and the B1 element at the 3′-end [22], and they are 147 nucleotides long and about 329,838 copies.

## 3. *Alu* and Human Genomic Diversity

*Alu* mobile elements were identified originally 30 years ago in the human DNA [25] and were named for an internal *AluI* restriction enzyme recognition site [26]. The sequence and structure analysis indicated that *Alu* elements were ancestrally derived from the 7SL RNA gene which is a component of the ribosomal complex [13]. They were present at 500,000 copies [27], and they recently have arisen to a copy number in excess of one million within the human genome [28]. The amplification of *Alu* elements is thought to occur by the reverse transcription of an *Alu*-derived RNA polymerase III transcript in a process called retrotransposition [1]. A self-priming mechanism of reverse transcription by the *Alu* RNAs has also been proposed [29]. Because *Alu* elements have no open reading frames, they use for their amplification the machinery and the exogenous enzymatic function of long interspersed nuclear elements (LINEs) [2, 30–32]. In addition, the poly(A) tails of LINEs and *Alu* elements are thought to be the common structural features that are involved in the competition of these mobile elements for the same enzymatic machinery for mobilization [33]. *Alu* sequences within the human genome can be divided into subfamilies based upon diagnostic mutations shared by subfamily members, and they appear to be of different genetic ages [34–39]. The earliest *Alu* elements were the J subfamily, followed by S subfamilies that include Sx, Sq, Sp, and Sc, and followed by the more recent Y subfamilies including Ya5 and Yb8 the most dominant in humans [11, 40, 41]. The young *Alu* elements provide new information about the genomic fossils for the study of human genetic diversity. The rate of *Alu* amplification is estimated to be of the order of one new *Alu* insertion in every 20 births [42, 43]. Homologous recombination between dispersed *Alu* elements might result in various genetic exchanges, including duplications, deletions, and translocation which could be a mechanism for the creation of genetic diversity in the human genome. The fixation of specific mobile element insertion sites in a population can be used as a distinct character for phylogenetic analysis and could be useful markers for studies of human population diversity and origins [44–47]. It has been reported that there have been about 5,000 lineage-specific insertions fixed in the human genome since their divergence [48, 49]. However, *Alu* insertion could also have negative consequences and could induce damage to the human genome.

## 4. *Alu*-Mediated Recombination and Insertional Mutagenesis Contribution to Human Diseases

Several genetic disorders can result from different types of mutations that arise following the insertion of an *Alu* retroelement. The human genome project hg18 identified 584 human reference-specific *Alu* insertions [43]. *Alu* insertion can influence the genome stability, and it accounts for 0.1% of all human genetic disorders [9] such as hereditary desmoid disease [50], cystic fibrosis [51], Dent's disease [52, 53], X-linked agammaglobulinemia [54–57], hemophilia A and B [58–60], autoimmune lymphoproliferative syndrome [61], Apert syndrome [62], neurofibromatosis type 1 [63], benign isolated glycerol kinase deficiency [64], hyper IgM with immunodeficiency syndrome [65], Menkes disease [66], Alstrom syndrome [67], retinitis pigmentosa [68], acholinesterasemia [69], autosomal dominant optic atrophy [70], hemolytic anemia [24], autosomal branchio-oto-renal syndrome [71], acute intermittent porphyria [72], mucolipidosis II [73], and several type of cancer [23, 74–77] to cite a few. There are several mechanisms by which *Alu* can alter genomic structure. In addition to the potential impact of *Alu* retroelement insertions in causing human diseases, their broad dispersion throughout the genome provides opportunity for unequal homologous recombination and cross-over. Recombination between *Alu* retroelements on the same chromosome results in either duplication or deletion of the sequences between the *Alus*. When the recombination occurs on different chromosomes, it leads to chromosomal translocations or rearrangements. Several human diseases have been reported to be associated with *Alu* recombination events such as Gaucher's disease [78], hypercholesterolemia [79–82], chronic granulomatous disease [83], *α*-thalassaemia [84, 85], diabetes [86], thrombophilia [87], hypobetalipoproteinemia [88], and spastic paraplegia type 11 [89].

The vast majority of *Alu* insertions that have led to human disease insert into coding exons, near the promoter/enhancer regions, or into introns relatively near an exon. *Alu* insertions contribute to disease by either altering the transcription of a gene by affecting its promoter (changing the methylation status or introducing an additional regulatory sequence) or disrupting a coding region, or disrupting the splicing of a gene. These mechanisms have been intensively discussed previously, and the reader is directed to several elegant reviews [11, 90–92]. Although *Alu* elements are broadly spread throughout the human genome, some genes, chromosomes, and regions seem to be more prone to disease-causing insertions than others. 

## 5. *Alu* RNA Accumulation Induces Age-Related Macular Degeneration (AMD)

*Alu* RNA expression and accumulation, rather than retrotransposition, insertion, or recombination per se, has been recently shown to be involved in the advanced “dry” age-related macular degeneration disease [10], the leading cause of blindness in elderly worldwide [10, 93]. This atrophic form, geographic atrophy (GA), involves alterations of pigment distribution, loss of RPE cells and photoreceptors and diminished retinal function due to an overall atrophy of the cells [94]. All studies confirm the strong age dependence of the disease, which likely arises from a complex interaction of metabolic, functional, genetic, and environmental factors [95–97]. Although the molecular mechanisms underpinning this disease are not completely understood, there is intriguing evidence that exogenous double-stranded RNA (dsRNA) can activate toll-like receptor-3- (TLR3-) mediated inflammatory and chemokine protein secretion and -induced RPE cell death [98–100]. TLR3 knockout mice are protected against RPE degeneration induced by exogenous dsRNAs [100]. The phenomenon observed in mouse model for AMD has led to the hypothesis that the activation of TLR3 by endogenous dsRNAs may cause AMD in humans. Kaneko and coworkers [10] detected abundant dsRNA immunoreactivity in the RPE from diseased but not normal human eyes. Sequence-independent amplification of these immunoprecipitated and isolated dsRNAs showed amplicons belongs to the *Alu *Sq subfamily (GenBank accession nos. HN176584 and HN176585). It has become clear that bidirectional transcription and dsRNA formation are more prevalent than had been previously thought [101–103]. *Alus* are capable of folding back to generate hairpin structures. Two close (<2 kb) *Alu* elements in opposite orientation might base pair leading to the formation of a long stable dsRNA and becoming a major target for adenosine deaminase acting on RNA (ADARs) A-to-I editing [104]. Although the precise role of RNA editing is still speculative, it might influence the stability of dsRNA and its nuclear retention [105–107]. *Alu* RNAs seem to be free and nonembedded polymerase III transcripts [10] and were accumulated mainly in the cytoplasm of RPE cells indicating that they might escape ADARs editing and nuclear retention. However, there are no available data, to the best of our knowledge, concerning ADARs and paraspeckle-associated complex activity in the RPE from GA compared to normal eye, and this area will undoubtedly need further investigations. Once in the cytoplasm, *Alus* should be cleaved by DICER1 since they have been shown to be substrates for DICER1. The RNase DICER1, micro RNA- (miRNA-) processing key enzyme, has been shown to be dramatically downregulated in the RPE from GA compared to normal eye which explains the accumulation of *Alu* RNAs [10]. Interestingly, DICER1 which is also expressed in the nucleus of RPE cells and its function (whether dicing *Alu* or not) as well as its nuclear expression levels in GA compared to normal eye are still unknown. In parallel experiment in mice, loss of Dicer1 induced B1/B2 (*Alu*-like elements) accumulation and RPE cell degeneration. Alternatively, this leads to additional biological questions such as in normal conditions where DICER1 is fully functional, what are the *Alu*- (or B1/B2-) cleaved products? How long are they? And what is (are) their biological function(s)? 

**Figure 2:**
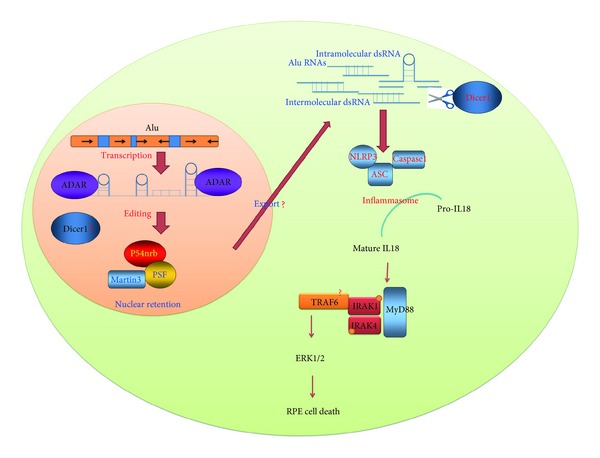
Model for the fate of *Alu* RNAs in the RPE from GA eye. *Alus* can form long duplex RNA structures and pose as targets for ADAR A-to-I RNA editing activity. The edited *Alu *RNAs may be bound by the paraspeckle that contains the nuclear proteins P54nrb, PSF and martin 3 and are expected to be retained on the nuclear matrix in normal eye. In diseased eye, DICER1 is dysregulated and *Alu *RNAs are exported and accumulated in the cytoplasm leading to the activation of NLRP3 inflammasome and MyD88, which in turn may activate ERK1/2 and induce RPE cell degeneration.

Unexpectedly and in contrast to exogenous dsRNAs, *Alus* induced RPE cell death independently of miRNA and TLR3 as well as a variety of other TLRs and RNA sensors [108]. *In vivo* and *in vitro* functional studies showed that *Alus* induced RPE cell death via innate immune sensing pathway and activated NLR family, pyrin domain containing 3 (NLRP3) inflammasome [108]. Activation of the NLRP3 inflammasome triggered activation of caspase-1 and induced maturation of interleukin-18 (IL-18) which in turn activated the myeloid differentiation primary response gene 88 (MyD88) pathway (phosphorylation of interleukin-1 receptor-associated kinase-1 and -4 (IRAK1 and IRAK4)) [108] (Figure 2). The effect of *Alu* RNA on RPE cell degeneration was mediated also via activation of extracellular signal-regulated kinase (ERK)1/2 MAPK [109]; however, the up- and downstream cascades are still unknown and further investigations are warranted. It is conceivable that ERK1/2 activation might be downstream of IL-18 and MyD88 [110–112], and several other potential *Alu*-mediated signaling pathways might be involved.

## 6. ADAR Gene Family and *Alu* RNA Editing

The adenosine deaminases acting on RNA (ADARs) are proteins that bind to double-stranded RNA and cause the modification of adenosine to inosine via a hydrolytic deamination reaction [113]. Editing of RNA from A to I in the coding regions of specific genes can lead to functional alterations of the protein product [114, 115], whereas editing of the noncoding regions may affect splicing, stability, or the translational efficiency of these target mRNAs [116, 117]. The precise role of RNA editing is still speculative, and ADAR may act as an antiviral defense mechanism against dsRNA viruses [118], or antagonize dsRNA subjected to the RNAi-mediated gene silencing pathway [119, 120], and/or against dsRNA formed by *Alu* repeat elements or by sense and antisense transcripts. 

Three ADAR family members have been identified [121–125], and they are conserved in their C-terminal deaminase region as well as in their double-stranded RNA-binding domains. Mammalian ADAR1 and ADAR2 are ubiquitously expressed in many tissues; however, ADAR3 is mainly expressed in the brain [126]. ADAR3 has been shown to contain both single- and double-stranded RNA-binding domains. The dsRBDs of ADARs resemble those of dsRNA-activated protein kinase PKR which is an interferon inducible involved in antiviral mechanisms [127, 128] as well as Drosha and Dicer, key enzymes involved in miRNA biogenesis [129]. The ADAR editing efficiency increases with longer dsRNA [130]. RNA secondary structural features consisting of hairpins containing mismatches, bulges, and loops are edited more selectively than completely base-paired duplex RNA. The editing efficiency depends also on the sequence context of nucleotides surrounding the adenosine moiety to be edited [131]. Intriguingly ADAR3 is not active on the other known substrates of ADAR1/2 or on long dsRNA *in vitro*. ADARs act as a dimer in mammals, and ADAR1 and 2 do not form heterodimers and must form homodimers to be active [132]; however, ADAR3 does not dimerize which explains its lack of activity. There are two isoforms of ADAR1, the longer ADAR1p150 which is expressed in the cytoplasm and the nucleus and the shorter ADAR1p110 which remains in the nucleus [133]. Both isoforms harbor a nuclear localization signal [134]. Both ADAR1 and ADAR2 are present in the nucleolar compartment and are translocated to the nucleoplasm upon the presence of an active editing substrate [135, 136]. They are upregulated by inflammation and in presence of mRNA rich in inosine [137]. The ADARs proteins as well as their dsRNA substrates that mediate the A-to-I editing are important, and both of them determine what will be the overall effect of RNA editing. 

*Alus* are the major targets of ADAR A-to-I editing [104, 138–141] because they create long hairpin structures for which ADARs can deaminate. A computational analysis showed that 88% of the A-to-I editing events were found to be located in the *Alus,* even though they only comprise 20% of the total length of transcripts [140], and the editing was found to be the most prevalent in the brain compared to other tissues [139]. One may ask a question whether the *Alu* hairpin structures upon editing become more stable or unstable (reduced in its double strandness)? Previous studies are inconsistent; Levanon's and Blow's groups [139, 141] indicated that the effect of editing is aimed at destabilization of *Alu* dsRNA; however, Athanasiadis et al. [104] suggested that the overall effect is to stabilize the *Alu* dsRNA and this area need further investigations. The next question is what are the functional and biological consequences of *Alu* editing by ADARs? As the authors mentioned previously, *Alu* editing by ADARs may regulate the transcriptional activities of *Alu* during cellular stress or affect processing, stability (destability), nuclear retention, and export of *Alu* RNAs. While there is no direct biochemical evidence for RNAi-mediated chromatin silencing in higher eukaryotes, there is hypothesis that in mammalian cells nuclear dsRNA can induce transcriptional gene silencing associated with DNA methylation [142]. Furthermore, recent studies indicate a direct connection of the involvement of ADARs in the RNAi gene silencing pathway [143].

### 6.1. Other Cellular Mechanisms That May Deal with *Alu *dsRNAs

More than twenty proteins harboring dsRNA-binding domains (DRBPs) have been identified, and there are several distinct ways in which dsRNAs might be detected and resolved. The nuclear factors associated with dsRNA (NFAR) [144–147], nuclear members of the DRBPs, may interact with *Alu* dsRNA, although *Alu* dsRNAs induce RPE cell degeneration independently of PKR [108] and NFARs are physically associated with PKR, and they may function in PKR-mediated signaling events in the cell [147]. *Alu* dsRNA may also interact with spermatid perinuclear RNA-binding protein (SPNR) which is expressed in several tissues including testis, ovary, and brain. Although SPNR protein expression is limited to testis, neurological defects in mice lacking SPNR function indicate other roles for SPNR outside spermatogenesis [148]. *Alu* dsRNA might be degraded by dsRNA-specific nucleases [149] or unwound by dsRNA helicases [150, 151]. The RNA helicase A (RHA) has two DRBPs and binds to dsRNA as well as to ssRNA and ssDNA through a carboxyl-terminal RGG box [152, 153]. Other nuclear members of DRBPs such as the negative regulatory element binding protein (NREBP) [154, 155] and kanadaptin [156, 157] may interact with *Alu* dsRNA, although their role is still speculative. We have shown that Dicer dysregulation induced *Alu* accumulation and cytotoxicity in RPE cells, but we cannot rule out a potential involvement of other cytoplasmic members of DRBPs such as protein activator of PKR (PACT) [158] and staufen [159], and further studies are needed to determine whether *Alu* dsRNA binds to these nuclear and cytoplasmic DRBPs and their biological relevance in normal and diseased eye.

## 7. Concluding Remarks

Repeat elements are landscape-determining components of our genome, and they are “hot spots” elements that can affect our health through at least two known different mechanisms: (1) self-propagation and retrotransposition and (2) accumulation and cytotoxicity. Still, several questions remain unresolved: why and how *Alu* RNAs accumulate in the RPE of GA patients? It is possible that chronic stress insults (oxidative stress, heat shock, viral infection, etc.) in combination with increasing age and senescence induce *Alu* RNA accumulation [160–163]. Another important question is: are *Alu* RNAs accumulated in other age-related neurodegenerative diseases? However, some studies have suggested that the central nervous system is a privileged environment for transposition. In addition, DICER1 and the fine tuning of the miRNA gene network have been shown to be crucial for neuronal integrity. Indeed, genetic ablation of DICER1 induces neurodegeneration via hyperphosphorylation of tau protein and activation of ERK1/2 [164, 165]. Furthermore, the NALP3 inflammasome has been shown to be involved in Alzheimer's disease (AD) [166]. Altered DICER1 and miRNA regulation have been shown to be involved in other neurodegenerative diseases such as Huntington's [167] and Parkinson's diseases [168]; however, the *Alu* RNA profiling has not been reported yet.

The new sequencing technologies combined with rigorous functional analyses are available to study the mobilome, and they will certainly yield more valuable insights into both functional properties of the genomic gems and disease pathogenesis.
